# A SARS-CoV-2 outbreak investigation at a storage and distribution centre in England: an assessment of worker- and workplace-related risk factors

**DOI:** 10.1038/s41598-024-60194-4

**Published:** 2024-04-26

**Authors:** Amber I. Raja, Gillian Nicholls, Matthew Coldwell, Karin van Veldhoven, Vince Sandys, Barry Atkinson, Ian Nicholls, Antony Spencer, Alice Graham, Hannah Higgins, Christina Atchison, Chris Keen, Tony Fletcher, Neil Pearce, Elizabeth B. Brickley, Yiqun Chen

**Affiliations:** 1 Health Equity Action Lab, Department of Infectious Disease Epidemiology, London School of Hygiene & Tropical Medicine, London, UK; 2 Science Division, Health and Safety Executive, Buxton, UK; 3 Department of Non-Communicable Disease Epidemiology, London School of Hygiene & Tropical Medicine, London, UK; 4 Research and Evaluation, UK Health Security Agency, Porton Down, Salisbury, UK; 5 Rapid Investigation Team, Field Services, UK Health Security Agency, Wellington House, London, UK; 6 Chemical and Environmental Effects Department, UK Health Security Agency, Chilton, UK; 7 Department of Medical Statistics, London School of Hygiene & Tropical Medicine, London, UK

**Keywords:** Infectious diseases, Risk factors

## Abstract

An outbreak of SARS-CoV-2 (1 March to 10 May 2021) with an attack rate of 26.5% among approximately 1150 workers at a storage and distribution centre in England prompted a multidisciplinary outbreak investigation (5 May to 6 August 2021), with the aim of better understanding worker- and workplace-related risk factors for viral transmission in the warehousing sector. Overall, environmental factors (e.g., ventilation, humidity and temperature) were assessed to be appropriate at the facility. Nevertheless, 39 (51.3%) surface samples from across the site tested positive for low/ very low levels of SARS-CoV-2 RNA (Ct value ≥ 32.0 for all). Among the study participants, of whom 35.6% were confirmed or suspected cases, 95.5% reported having received COVID-19 prevention training, 100.0% reported handwashing, and 80.0% reported use of face coverings at work. Notably, 43.9% and 19.0% reported working with a symptomatic and a positive contact respectively. Furthermore, 80.5% and 46.3% had concerns regarding reduction in their income and future unemployment, respectively, due to self-isolation. The findings of this study suggest that, in addition to targeted workplace infection control measures and tailored work area specific risk assessments, an enhanced and equitable sick leave policy may help limit presenteeism and viral transmission in large workplaces.

## Introduction

The coronavirus disease 2019 (COVID-19) pandemic led to major shifts in our approach to work. During the height of the pandemic, non-essential work sectors in the United Kingdom (UK) were instructed to adopt a work-from-home policy by the government^1^. However, essential work sectors, including storage and distribution, were instructed to remain on-site and operational, putting essential workers at an increased risk of exposure to severe acute respiratory syndrome coronavirus 2 (SARS-CoV-2) and the development of COVID-19 compared to non-essential workers^2–7^. An analysis of COVID-19 workplace outbreaks across England between May and October 2020 found that warehousing workplaces, including storage and distribution centres, had some of the highest outbreak rates, second only to manufacturers and packers of food^2^. This has also been reported outside of the UK, with warehousing consistently ranked amongst work sectors with the highest incidences of COVID-19 in the United States^8–10^. Despite growing reliance on storage and distribution workplaces during and after the pandemic, warehouse worker pay is lower now than in 1990, after adjusting for inflation^11^. Additionally, although the UK has statutory sick pay policies in place, which allow eligible workers, including agency workers, to receive pay if they are unable to work due to sickness or injury, the policy in 2021 was equivalent to approximately 11 h of work per week (for a maximum of 2 weeks for sickness due to SAR-CoV-2) at minimum wage^12,13^. The research literature indicates an increase in COVID-19 outbreaks within warehousing workplaces; however, there has been a notable absence of comprehensive field studies, with direct workplace observations or real-time environmental measurements conducted close to the time of outbreaks, to understand the transmission risk factors in warehousing workplace settings. The lack of data hampers our ability to assess SARS-CoV-2 transmission risk factors in these essential workplaces and to inform effective mitigation strategies. Further research on warehousing workplace- and worker-related risk factors is needed.

**Figure 1:**
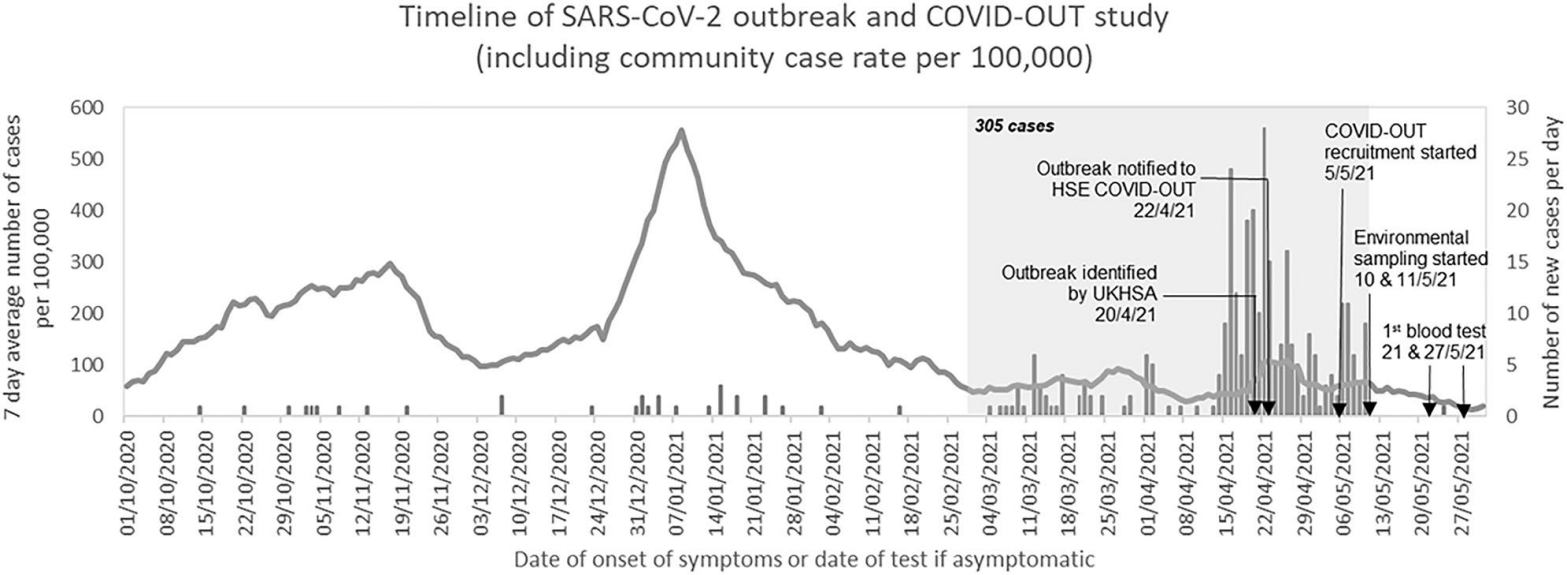
Timeline and epidemiological curve of a SARS-CoV-2 outbreak and the COVID-OUT investigation in a UK storage and distribution site. Arrows indicate key dates of the outbreak and the COVID-OUT study. The line chart represents the 7-day case rate for the lower tier local authority area (LTLA) of the site (contains public sector information licensed under the Open Government Licence v3.0 from 18; the date of download: 18 August 2021). The bars represent the number of new COVID-19 cases reported by the company. The grey box indicates the outbreak period (1 March to 10 May 2021), during which 305 acute SARS-CoV-2 infections were reported by the company. Abbreviations: UK Health Security Agency (UKHSA), Health and Safety Executive (HSE).

The COVID-OUT (COVID-19 Outbreak investigation to Understand Transmission) study, part of the UK PROTECT COVID-19 National Core Study^14^, aimed to understand outbreaks of SARS-CoV-2 and transmission risks across different work sectors^15^ using a multidisciplinary approach of environmental assessments and worker participation to systematically evaluate workplace outbreaks. A data collection framework was developed to cover a wide range of potential risk factors for investigation, including at the individual level, at the workplace environmental level and at the worker population level. Here, we report an investigation of an outbreak of SARS-CoV-2 infections, with an attack rate of 26.5%, occurring between 1 March and 10 May 2021 among approximately 1150 workers at a storage and distribution centre in England, UK. The outbreak took place during the phased exit from the third national lockdown (8 March to 19 July 2021^16^). Case rates of SARS-CoV-2 in the local area had been decreasing since mid-January 2021 (Fig. 1), in line with national trends^17,18^. An initial investigation (unpublished internal report) conducted by UK Health Security Agency (UKHSA; formerly known as Public Health England, PHE), identified the Alpha variant of SARS-CoV-2 (lineage: B.1.1.7) among cases from the outbreak. The subsequent investigation conducted by the COVID-OUT study between 5 May and 6 August 2021 assessed risk factors that may have contributed to the transmission of SARS-CoV-2 at this workplace, including those associated with worker behaviours and the workplace.

## Methods

From October 2020, the UKHSA Health Protection Team (HPT) was in contact with the storage and distribution centre due to a steady number of SARS-CoV-2 cases reported at the site (~ 6 cases/month). Between 1 March and 20 April 2021 (inclusive), the number of confirmed SARS-CoV-2 cases totalled 146 and was declared an outbreak by the UKHSA HPT on 20 April 2021 who subsequently notified the COVID-OUT team via the Health and Safety Executive (HSE) on 22 April 2021 (Fig. 1). Following this notification, the COVID-OUT team undertook a follow-up investigation from 5 May to 6 August 2021, using a previously described protocol^15^. Ethical approval was provided by the National Health Service (NHS) North East Research Ethics Committee (Reference 20/NE/0282). Informed consent was obtained from all study participants. All methods were carried out in accordance with relevant guidelines and regulations.

**Figure 2:**
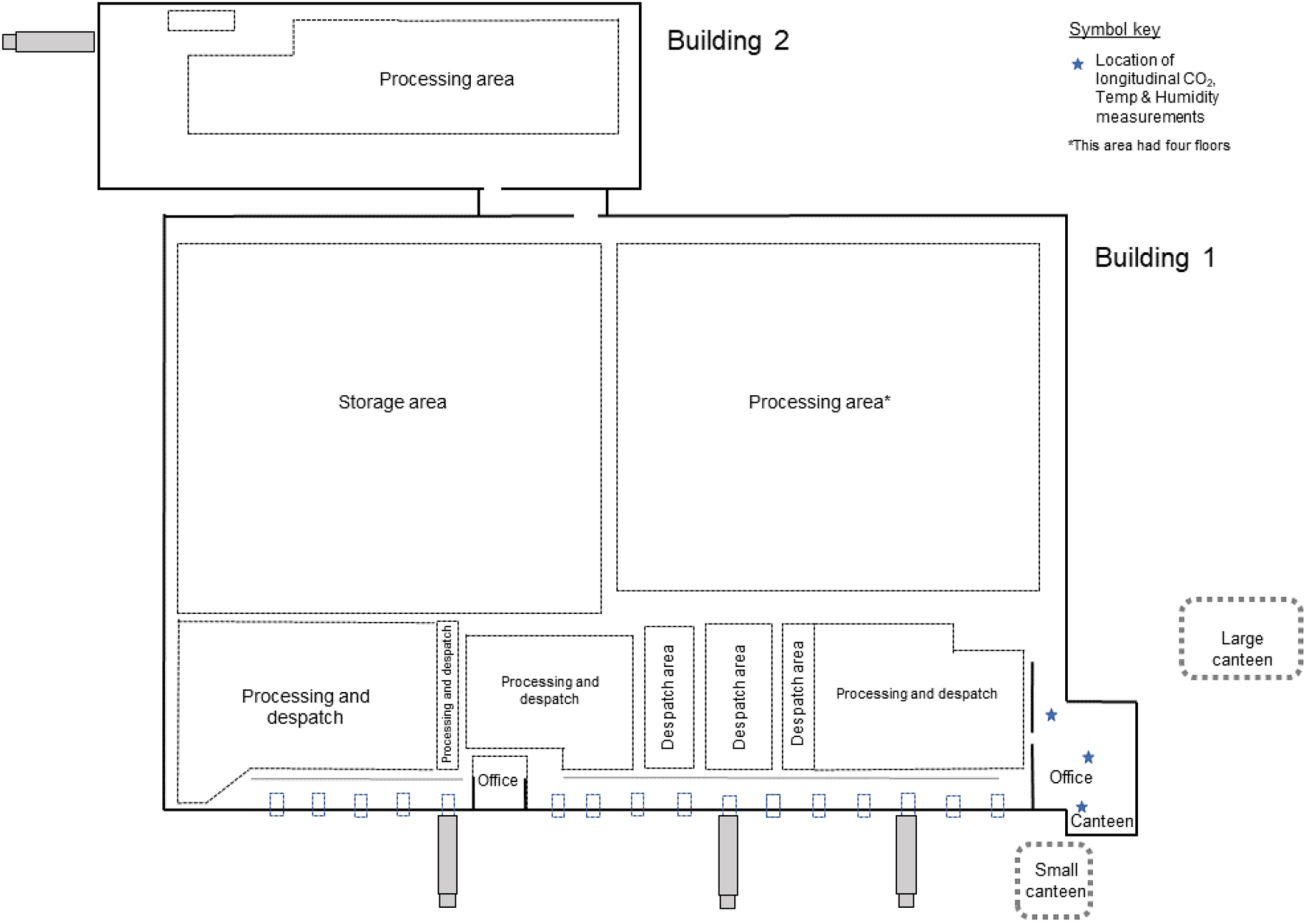
Floor plan of the storage and distribution site. Site divided into two buildings. Building 1 had an open plan layout comprising a large storage area, a large processing area with a mezzanine level (e.g., location of lockers) and four floors, and multiple small processing and dispatch areas dedicated to specific vendors. Welfare areas (e.g., canteen and toilets) are also located in Building 1, as well as offices located on the ground floor and a floor above the canteen. Two temporary canteens erected outside Building 1. Building 2 comprised a processing area, as well as a mezzanine level with lockers and a product spot-clean area. Abbreviations: Carbon dioxide (CO_2_), Temperature (Temp).

An environmental assessment was conducted by COVID-OUT occupational hygienists on 11 May 2021 following a published data collection framework^19^, which included collecting information on the building layout, ventilation, temperature, humidity, air movement and noise levels, as well as workforce information (e.g., shift patterns) and worker observations (e.g., adherence to infection control measures and worker interactions). Spot measurements of carbon dioxide (CO_2_; used as a proxy for ventilation in indoor spaces), humidity and temperature were taken on the day of the environmental assessment in various locations around the site, as well as being taken longitudinally in three locations (as noted in Fig. 2). Longitudinal readings were taken between 11 and 26 May 2021 for CO_2_ (logged every minute), temperature and humidity (logged every 2 min). Inadequate ventilation was indicated by CO_2_ concentrations of 1500 ppm or more^20^.

Surface sampling for SARS-CoV-2 viral RNA was conducted on 11 May 2021 according to a published protocol^15^, and as previously described^21^. Collected samples were analysed using the CerTest Biotec Viasure (Zaragoza, Spain) two-target N (nucleocapsid) and ORF1ab (open reading frame 1 a and b) assay. Confirmed positive samples were those with both replicates testing positive for at least one target, and suspected positive samples were those with a single replicate testing positive for at least one target. The limit of detection for the assay, as defined by the manufacturer, was a cycling threshold (Ct) value of 38.0. Samples with Ct values of ≤ 35.0 were further analysed by whole genome sequencing (WGS).

From 5 to 27 May 2021, all workers from the site were invited to participate in the study via email (i.e., sent to workers by the employer on behalf of the COVID-OUT study team) and via informational leaflets distributed in the canteen and during the initial on-site study visits. As previously described^15,22–25^, participants were asked to complete a detailed questionnaire at baseline, collecting information on COVID-19 symptoms, SARS-CoV-2 infection history, and potential risk factors, and three shorter follow-up questionnaires at weeks two, three and six (for full baseline and follow-up questionnaires, see:^26^). To mitigate the potential for social desirability bias, the questionnaire data were self-reported and collected online; behaviours that could be perceived as undesirable were presented as categorical (e.g., never, less than half the time, more than half the time, or nearly all the time) rather than dichotomous choices, as appropriate.

Participants also underwent SARS-CoV-2 antigen and antibody testing, as previously described^15,22–25^. Viral RNA testing was conducted on self-collected nose and throat swabs at baseline and weeks two and three using the Roche cobas® SARS-CoV-2 assay. Samples yielding Ct values of ≤ 35.0 were assessed for WGS. Antibody testing was conducted on blood samples collected by phlebotomists at baseline and week six, using the Roche Elecsys® (Basel, Switzerland) Anti-SARS-CoV-2 S (spike) and N binding assays. Confirmed cases were defined as participants who presented during the outbreak (1 March to 10 May 2021) with: (i) real-time polymerase chain reaction (RT-PCR) evidence of a SARS-CoV-2 infection, (ii) N-specific seroconversion, or (iii) self-reporting of a positive test (i.e., by RT-PCR or lateral flow device [LFD]) with positive N antibody results. Suspected cases were defined as participants who had no positive RT-PCR or N antibody results from the COVID-OUT testing but who presented during the outbreak period with: (i) a self-reported positive test (i.e., by RT-PCR or LFD) or (ii) symptoms consistent with COVID-19 defined as: (a) acute onset of fever (> 37.8 °C) and new continuous cough or (b) acute onset of any three or more symptoms of fever (> 37.8 °C), cough, shortness of breath, loss of taste or smell, runny nose, fatigue, sore throat, muscle or body aches, headache, nausea or vomiting, and/or diarrhoea.

An epidemiological curve was produced based on the anonymised case list provided to the COVID-OUT team by the UKHSA HPT, and covered the period from 14 October 2020 to 24 May 2021. The case list was constructed by the UKHSA HPT with input from the workplace, and provided the date of symptom onset or test date if asymptomatic. The epidemiological curve also presents the seven-day case rate for the local community, based on publicly available data for the Lower Tier Local Authority (LTLA) of the site^18^, to enable visual comparison with the workplace case list. Data analysis and summary figures were created using Stata/SE (version 17.0) and R (version 3.6.2).

### Disclosure

The contents of this paper, including any opinions and/or conclusions expressed, are those of the authors alone and do not necessarily reflect Health and Safety Executive or UK Health Security Agency policy.

## Results

### Environmental risk assessment

The storage and distribution centre had a total workforce of approximately 1150 workers on various shift patterns, half of whom were agency workers. The LTLA of the site had seen peaks in the seven-day case rate of 298 and 555 cases per 100,000 in mid-November 2020 and mid-January 2021, respectively. During these periods, the site had reported cases but not to a level that would constitute an outbreak (Fig. 1). The outbreak started on 1 March 2021. By 10 May 2021, the overall attack rate at this site was 26.5% (305/1150) according to the company’s records on the number of test-positive cases in the workforce. During the course of this outbreak, the LTLA seven-day case rate fluctuated slightly but generally remained below 100 cases per 100,000.

The storage and distribution centre was composed of two buildings (combined size of approximately 33,530m^2^; Fig. 2). Building 1 (size = 29,350m^2^; ~ 150–200 workers per shift) had an open plan layout comprising a large storage area, a large processing area with a mezzanine level (e.g., location of some personnel lockers) and four floors, and multiple small processing and dispatch areas dedicated to specific vendors. Welfare areas (e.g., canteen and toilets) were also located in Building 1, as well as offices located on the ground floor and first floor above the canteen. Building 2 (size = 4180m^2^; ~ 100 workers per shift) also had an open plan layout for processing, as well as a mezzanine level with lockers and a product spot-cleaning area.

A risk assessment had been conducted by site management at the start of the pandemic, and a number of measures were introduced prior to the outbreak, including signs and floor markings throughout the buildings to promote 2m social distancing, maximum occupancy limits for canteens and toilets, a traffic light system in place for toilets, enhanced cleaning measures, provision of hand sanitiser throughout the workplace, dividers installed in work areas where 2m social distancing could not be maintained, and policies to encourage office workers to work-from-home. The use of face coverings was required when walking between different areas of the site, but not whilst at workstations. Two additional canteens and additional toilet facilities were erected outside Building 1 to further facilitate social distancing.

The site management also introduced separate entry and exit points and staggered start times, which reduced the number of workers entering the premises at any given time and allowed for staggered break times. All workers entered the site through a single access point in Building 1, to reach the clocking-in area in this building before passing through a turnstile to access the main work area. The clocking-in area was noted to be a potential pinch point at the start of shifts, though no overcrowding was observed in this area during the COVID-OUT environmental assessment site visits. Workers from Building 2 had to pass through Building 1 in order to access their work areas. There were one-way systems in place throughout Building 1, including in stairwells and corridors. However, these areas were not always of sufficient width to maintain 2m social distancing, though any contact here would be transient. To exit the main work areas, workers passed through a security station in Building 1 before exiting the building through the canteen.

Work areas were arranged to allow for social distancing, and most were fitted with dividers. In the main processing area in Building 1, workers on parallel processing lines were screened from adjacent workers but not to those working on the opposite side of the conveyer belt; however, the width of the conveyor belt was > 2m, allowing more than sufficient space for social distancing. Workers spent most of their time in the same work area, with little movement to other work areas. In the product spot-cleaning area in Building 2, it was not always possible to maintain social distancing; however, this area had a maximum occupancy of six workers, dividers were fitted and downdraught benches, which draw air away from the workers’ breathing zone and expel the air externally, were in use. There were only a limited number of workers in the offices, largely due to the work-from-home policy. COVID-OUT occupational hygienists observed good compliance in maintaining social distancing during the environmental assessment. The company reported to the occupational hygienists that their own investigations did not indicate any obvious clustering of cases related to specific work areas.

The worksite initiated two rounds of RT-PCR testing for workers between 22 April and 8 May 2021, independently of the COVID-OUT study. On the week commencing 10 May 2021, one of the additional canteens was re-assigned as an LFD testing unit, and weekly LFD testing of all workers was introduced, initially with supervised testing on-site, with the aim to move towards workers self-testing at home. The site remained operational throughout the outbreak; however, some volume of work was diverted to other facilities to reduce the number of workers on-site. Some infection control measures were revised following the outbreak, notably increasing cleaning and mandating the use of face coverings at all times across the site, for which the company provided face coverings (type not specified). Occupational hygienists observed a high level of compliance in the wearing of face coverings during the site visit.

Forced mechanical ventilation systems were present in Building 1’s main warehouse area, offices and welfare areas (i.e., toilets and canteen). The ventilation system was operated by a building management system on a continuous basis and used both fresh and recirculated air. It recirculated up to 95% of extracted air during the colder months and introduced up to 100% fresh air during the warmer months. The air was passed through G4 filtration, which was replaced at least every three months. The company indicated that at the time of the outbreak the ventilation system was likely to be using 100% fresh air. In Building 2, there was no forced mechanical ventilation; however, because the two buildings were connected to each other, it is likely that some airflow was introduced into Building 2 from the ventilation system in Building 1. In the processing areas of both buildings there were a number of roller shutter doors and fire doors that could be opened to increase airflow at the workers’ discretion. The fire doors were recently modified to allow them to be left open by workers, if desired, but would automatically close during a fire alarm to maintain fire safety. Some of the windows in the canteen area and offices of Building 1 were left open. Windows in the canteen had been fitted with brackets, so that windows were permanently left ajar, although it was not confirmed whether these were fitted before the outbreak. In the temporary canteens, access doors at the front and back were open as were several windows located along the length of the structure.

Spot measurements of CO_2_ in office and warehouse areas taken during the site visit were typically no more than 400–600 ppm indicating sufficient ventilation. Longitudinal CO_2_, temperature and relative humidity measurements were taken in the conference room, HR office and the main office in Building 1. Levels of CO_2_ in the conference room did not exceed 700 ppm. Regular cleaning and only occasional use of the conference room was reported (e.g., a total of 12 meetings over 11 days, ≤ 2 h and ≤ 4 occupants per meeting). CO_2_ levels in the HR office did not exceed 800 ppm; occupancy was typically no more than 3 people. These levels indicated adequate ventilation in these areas. An instrument error meant that no data was collected from the main office. The mean relative humidity in the conference room was 48.8% (range 32.5–50.1%), the HR office was 42.8% (range 34.0–50.0%) and the main office was 38.6% (range 28.9–45.2%). The mean temperature in the conference room was 21.1 °C (range 15.7–23.3 °C), the HR office was 21.9 °C (range 19.6–26.2 °C) and the main office was 21.6 °C (range 19.0–24.8 °C). Occupational hygienists reported that noise levels in the processing areas of both buildings were not a barrier to communication.

### Viral surface sampling

The surfaces of work (e.g., benches, computers) and communal areas (e.g., lockers, vending machines), and high-touch surfaces (e.g., tap handles) were sampled for viral RNA. A total of 76 samples were collected from across the site; with 25 (32.9%) samples confirmed positive for SARS-CoV-2 RNA and 14 (18.4%) identified as suspected positive (Table 1). The level of RNA detected in 90.8% (69/76) of samples was very low (Ct value ≥ 35.0) or undetectable. Although, seven samples from across the site produced Ct values between 32.9 and 35.0 (Table S1). Of note, multiple positive and suspected positive samples were identified in distinct areas across the site, including work areas (e.g., a packing bench on the 3rd floor, where 3/4 samples were confirmed positives and all three positive samples had Ct values < 35.0) and communal areas (e.g., a breakout area with 5/7 samples confirmed positive). The seven samples with Ct values ≤ 35.0 were sent for WGS but did not pass the quality control parameters, indicating highly degraded genomic material.

**Table 1:** SARS-CoV-2 RNA results of 76 surface samples taken from various locations following an outbreak at a storage and distribution centre—England, UK.

RT-PCR results (From a total of 76 samples)			Level of RNA (Based on Ct value)		
Confirmed positive	Suspected positive	Negative	Moderate-high (Ct < 32.0)	Low (Ct 32.0–34.9)	Very low-none (Ct ≥ 35.0^a^)
25 (32.9%)	14 (18.4%)	37(48.7%)	0 (0.0%)	7 (9.2%)	69 (90.8%)

^a^Includes 37 samples with no SARS-CoV-2 RNA detected.

Abbreviations: Severe acute respiratory syndrome coronavirus 2 (SARS-CoV-2), Ribonucleic acid (RNA), Real-time polymerase chain reaction (RT-PCR), Crossing threshold (Ct).

### Participant questionnaire responses

Forty-five workers (60% female; mean age 35.7 years, range 18–70; Table 2) participated in the COVID-OUT study (Fig. S1). The estimated response rate was 3.9%, which may be an underestimate as the proportion of the total workforce (approximately 1150 workers across various shift patterns) who were on-site during study recruitment could not be confirmed. Of these, 13 participants (76.9% female; mean age 29.2 years, range 18–43) were confirmed cases who self-reported positive SARS-CoV-2 tests during the course of the outbreak (1 March to 10 May 2021) and tested positive for both N- and S-specific antibodies against SARS-CoV-2 during the COVID-OUT study. A further three participants (100% female; mean age 49.3 years, range 28–62) were suspected cases, two of whom self-reported positive SARS-CoV-2 tests and one who reported three or more COVID-19 symptoms without a positive SARS-CoV-2 test during the COVID-OUT study. The remaining 29 participants (48.3% female; mean age 37.2 years, range 21–70) were identified as non-cases.

**Table 2:** Participants' work-related responses in the baseline COVID-OUT study questionnaire.

		All (N = 45)	Non-cases (N = 29)	All Cases (N = 16)	Confirmed cases (N = 13)	Suspected cases (N = 3)
Sex	Male	18 (40.0)	15 (51.7)	3 (18.8)	3 (23.1)	0 (0.0)
	Female	27 (60.0)	14 (48.3)	13 (81.3)	10 (76.9)	3 (100.0)
Age	Mean (range), years	35.7 (18–70)	37.2 (21–70)	32.9 (18–62)	29.2 (18–43)	49.3 (28–62)
Work factors						
Work area	All work areas	5 (12.5)	5 (20.0)	0 (0.0)	0 (0.0)	0 (0.0)
	Office	3 (7.5)	0 (0.0)	3 (20.0)	2 (16.7)	1 (33.3)
	Warehouse	32 (80.0)	20 (80.0)	12 (80.0)	10 (83.3)	2 (66.7)
	Missing	5	4	1	1	0
Work mainly indoors	No	6 (14.6)	4 (15.4)	2 (13.3)	1 (8.3)	1 (33.3)
	Yes	35 (85.4)	22 (84.6)	13 (86.7)	11 (91.7)	2 (66.7)
	Missing	4	3	1	1	0
No. of contacts whilst working indoors, if applicable	Alone	3 (8.6)	1 (4.6)	2 (15.4)	2 (18.2)	0 (0.0)
	1–5	7 (20.0)	1 (4.6)	6 (46.2)	6 (54.5)	0 (0.0)
	6 or more	25 (71.4)	20 (90.1)	5 (38.5)	3 (27.3)	2 (100.0)
	Missing	4	3	1	1	0
Indoor fresh air, if applicable	No	7 (20.0)	4 (18.2)	3 (23.1)	3 (27.3)	0 (0.0)
	Yes, mechanical	15 (42.9)	10 (45.5)	5 (38.5)	3 (27.3)	2 (100.0)
	Yes, by mechanical + by opening windows/doors	3 (8.6)	3 (13.6)	0 (0.0)	0 (0.0)	0 (0.0)
	Yes, opening window/door	6 (17.1)	2 (9.1)	4 (30.8)	4 (36.4)	0 (0.0)
	Yes, other	1 (2.9)	1 (4.6)	0 (0.0)	0 (0.0)	0 (0.0)
	Don’t know	3 (8.6)	2 (9.1)	1 (7.7)	1 (9.1)	0 (0.0)
	Missing	4	3	1	1	0
Close or physical contact with colleagues or members of the public	No	30 (71.4)	17 (63.0)	13 (86.7)	11 (91.7)	2 (66.7)
	Yes, co-worker	11 (26.2)	9 (33.3)	2 (13.3)	1 (8.3)	1 (33.3)
	Yes, co-worker and public	1 (2.4)	1 (3.7)	0 (0.0)	0 (0.0)	0 (0.0)
	Missing	3	2	1	1	0
Divider between colleagues (if close/physical contact reported)	No	2 (22.2)	2 (25.0)	0 (0.0)	0 (0.0)	0 (0.0)
	Yes	7 (77.8)	6 (75.0)	1 (100.0)	0 (0.0)	1 (100.0)
	Not applicable	30	17	13	11	2
	Missing	6	4	2	2	0
Socially distanced from colleagues	Rarely	1 (2.4)	0 (0.0)	1 (6.7)	0 (0.0)	1 (33.3)
	Sometimes	2 (4.9)	1 (3.9)	1 (6.7)	1 (8.3)	0 (0.0)
	Mostly	35 (85.4)	23 (88.5)	12 (80.0)	10 (83.3)	2 (66.7)
	Always	3 (7.3)	2 (7.7)	1 (6.7)	1 (8.3)	0 (0.0)
	Missing	4	3	1	1	0
Need to talk loudly or lean in	No	19 (45.2)	12 (44.4)	7 (46.7)	5 (41.7)	2 (66.7)
	Yes	23 (54.8)	15 (55.6)	8 (53.3)	7 (58.3)	1 (33.3)
	Missing	3	2	1	1	0

Participants reported working in offices (7.5%), the warehouse (80.0%) and in all work areas (12.5%; Table 2), with the majority working as warehouse operatives (64.1%; 25/39; Standard Occupational Classification [SOC] code: 9252) and managers in logistics (12.8%; 5/39; SOC code:1243). Thirty-five (85.4%; 35/41) participants reported working mainly indoors, of whom 71.4% (25/35) reported access to fresh air whilst working, 20.0% (7/35) reported no access to fresh air and 8.6% (3/35) reported that they did not know. Most respondents working mainly indoors reported working with six or more contacts whilst indoors (71.4%; 25/35). Over a quarter (28.6%; 12/42) of respondents indicated that their work required close or physical contact and 2.4% (1/41) reported that they were rarely socially distanced from colleagues. The majority of participants (54.8%; 23/42) felt they regularly had to talk loudly or lean-in to listen and speak to people at work, and 77.8% (7/9), for whom it was applicable, reported that there were no dividers between them and their colleagues. Although 81.4% (35/43) of participants reported having permanent work contracts, the employment-related risks of self-isolation due to COVID-19 appeared to be a significant concern. Nearly half (47.5%, 19/40) indicated that they thought their current pay would decrease, with another 10.0% indicating that they thought their pay would become zero if they self-isolated (Table 3). When asked about the future, 80.5% (33/41) were worried that self-isolation could have deleterious consequences for their future income, and 46.3% (19/41) had concerns about the risk of unemployment.

**Table 3:** Participants' employment-related responses in the baseline COVID-OUT study questionnaire.

		All (N = 45)	Non-cases (N = 29)	All Cases (N = 16)	Confirmed cases (N = 13)	Suspected cases (N = 3)
Employment contract	Permanent	35 (81.4)	23 (82.1)	12 (80.0)	10 (83.3)	2 (66.7)
	More than a year fixed term	2 (4.7)	1 (3.6)	1 (6.7)	0 (0.0)	1 (33.3)
	Zero hours contract	6 (14.0)	4 (14.3)	2 (13.3)	2 (16.7)	0 (0.0)
	Missing	2	1	1	1	0
Thinks pay would change due to self-isolation	No change	7 (17.5)	3 (12.5)	4 (25.0)	3 (23.1)	1 (33.3)
	Decrease	19 (47.5)	11 (45.8)	8 (50.0)	7 (53.9)	1 (33.3)
	Become zero	4 (10.0)	3 (12.5)	1 (6.3)	1 (7.7)	0 (0.0)
	Don’t know	10 (25.0)	7 (29.2)	3 (18.8)	2 (15.4)	1 (33.3)
	Missing	5	5	0	0	0
Thinks pay would change due to 2-week work closure	No change	2 (4.9)	1 (4.0)	1 (6.3)	0 (0.0)	1 (33.3)
	Decrease	13 (31.7)	7 (28.0)	6 (37.5)	5 (38.5)	1 (33.3)
	Become zero	7 (17.1)	4 (16.0)	3 (18.8)	3 (23.1)	0 (0.0)
	Don’t know	19 (46.3)	13 (52.0)	6 (37.5)	5 (38.5)	1 (33.3)
	Missing	4	4	0	0	0
Has concerns about income reduction in future due to self-isolation	No	5 (12.2)	3 (12.0)	2 (12.5)	2 (15.4)	0 (0.0)
	Yes	33 (80.5)	20 (80.0)	13 (81.3)	11 (84.6)	2 (66.7)
	Not sure	3 (7.3)	2 (8.0)	1 (6.3)	0 (0.0)	1 (33.3)
	Missing	4	4	0	0	0
Has concerns about unemployment in future due to self-isolation	No	18 (43.9)	11 (44.0)	7 (43.8)	5 (38.5)	2 (66.7)
	Yes	19 (46.3)	11 (44.0)	8 (50.0)	7 (53.8)	1 (33.3)
	Not sure	4 (9.8)	3 (12.0)	1 (6.3)	1 (7.7)	0 (0.0)
	Missing	4	4	0	0	0

**Figure 3:**
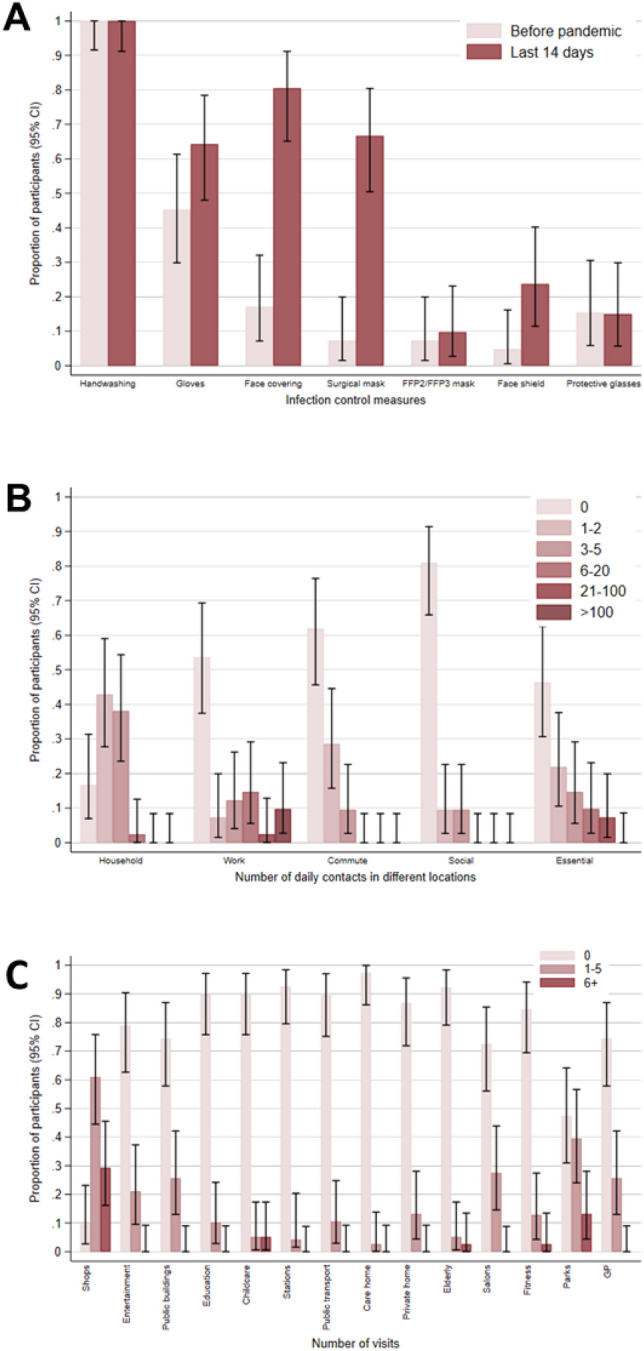
Baseline questionnaire responses of participants (N = 45) from a storage and distribution site. (**A**) Proportion of participants reporting infection control measures at workplace before the pandemic and within 14 days prior to completing the questionnaire. The following data were missing: handwashing before pandemic (n = 3) and in last 14 days (n = 5), gloves before pandemic (n = 3) and in last 14 days (n = 3), face covering before pandemic (n = 4) and in last 14 days (n = 4), surgical mask before pandemic (n = 4) and in last 14 days (n = 3), FFP2/ FFP3 mask before pandemic (n = 4) and in last 14 days (n = 4), face shield in pandemic (n = 3) and in last 14 days (n = 7), protective glasses before pandemic (n = 6) and in last 14 days (n = 5), (**B**) Proportion of participants reporting their daily number of contacts in different locations within the last 14 days of completing the questionnaire. The following data were missing: household contacts (n = 3), work contacts (n = 4), commute contacts (n = 3), social contacts (n = 3), and essential contacts (n = 4). (**C**) Proportion of participants reporting the number of visits they had made to different locations within the last 14 days of completing the questionnaire. The following data were missing: visit to shops (n = 4), visit to entertainment venue (n = 7), visit to public buildings (n = 6), visit to education facility (n = 6), visit to childcare (n = 6), visit to stations (n = 5), visit to public transport (n = 7), visit to care home (n = 7), visit to socialise in a private home (n = 7), visit to care for vulnerable adult in a private home (n = 6), visit to salons (n = 5), visit to fitness facility (n = 6), visit to parks (n = 7), visit to medical facility (n = 6). Error bars represent 95% confidence intervals (CI).

The questionnaire also evaluated participants’ COVID-19-related preventive behaviours and their contact patterns inside and outside of work. Almost all (95.5%; 42/44) reported having received workplace training (i.e., reading guidance and/or formal training) about preventing COVID-19 transmission (Table 4). Overall, participants reported high uptake of infection control measures within 14 days of completing the baseline questionnaire, including increased use of face coverings and surgical masks compared to the pre-pandemic period (Fig. 3A). However, use of FFP2/FFP3 masks remained low and did not materially increase, with only minimal uptake from the pre-pandemic period (7.3%; 3/41) to within 14 days of completing the baseline questionnaire (9.8%; 4/41). In contrast, self-reported hand hygiene was high, with all (40/40) participants reporting handwashing at work within the past 14 days, 97.6% (41/42) reporting the presence of hand washing/sanitising facilities, and 92.7% (38/41) noting informational signage encouraging good hand hygiene practice. Overall, participants reported higher numbers of close contacts at work and during essential activities (e.g., food shopping or visiting GP), with numbers at home, whilst commuting, and during social activities (e.g., going to restaurants) mostly limited to five or fewer close contacts (Fig. 3B). The most visited venues outside of work were retail facilities and parks (Fig. 3C). The majority of participants (73.3%; 33/45) reported travelling to work by car within the 14 days prior to completing the questionnaire, with no use of public (e.g., buses, trains) or company transport being reported and only minimal use of car sharing (11.1%; 5/45).

**Table 4:** Participants' COVID-19 prevention-related responses in the baseline COVID-OUT study questionnaire.

		All (N = 45)	Non-cases (N = 29)	All cases (N = 16)	Confirmed cases (N = 13)	Suspected cases (N = 3)
Vaccinated prior to outbreak	No	43 (97.7)	27 (96.4)	16 (100.0)	13 (100.0)	3 (100.0)
	Yes	1 (2.3)	1 (3.6)	0 (0.0)	0 (0.0)	0 (0.0)
	Missing	1	1	0	0	0
No. of vaccine doses prior to outbreak	1	1 (100.0)	1 (100.0)	0 (0.0)	0 (0.0)	0 (0.0)
	2	0 (0.0)	0 (0.0)	0 (0.0)	0 (0.0)	0 (0.0)
	Not applicable	43	27	16	13	3
	Missing	1	1	0	0	0
Vaccinated after start of outbreak	No	26 (60.5)	15 (55.6)	11 (68.8)	9 (69.2)	2 (66.7)
	Yes	17 (39.5)	12 (44.4)	5 (31.3)	4 (30.8)	1 (33.3)
	Not applicable	1	1	0	0	0
	Missing	1	1	0	0	0
Stopped work when had symptoms	No	2 (12.5)	2 (25.0)	0 (0.0)	0 (0.0)	0 (0.0)
	Yes, sometimes	1 (6.3)	1 (12.5)	0 (0.0)	0 (0.0)	0 (0.0)
	Yes, always	13 (81.3)	5 (62.5)	8 (100.0)	7 (100.0)	1 (100.0)
	Missing	29	21	8	6	2
COVID-19 training at work	No	2 (4.5)	2 (7.1)	0 (0.0)	0 (0.0)	0 (0.0)
	Yes	42 (95.5)	26 (92.9)	16 (100.0)	13 (100.0)	3 (100.0)
	Missing	1	1	0	0	0
Hand washing or sanitising facilities at work	No	1 (2.4)	0 (0.0)	1 (6.7)	1 (8.3)	0 (0.0)
	Yes	41 (97.6)	27 (100.0)	14 (93.3)	11 (91.7)	3 (100.0)
	Missing	3	2	1	1	0
Good hand hygiene practice signage at work	No	3 (7.3)	1 (3.9)	2 (13.3)	1 (8.3)	1 (33.3)
	Yes	38 (92.7)	25 (96.2)	13 (86.7)	11 (91.7)	2 (66.7)
	Missing	4	3	1	1	0
Close contact with symptom(s)	No	21 (51.2)	11 (42.3)	10 (66.7)	9 (75.0)	1 (33.3)
	Yes, live with	2 (4.9)	2 (7.7)	0 (0.0)	0 (0.0)	0 (0.0)
	Yes, work with	3 (7.3)	3 (11.5)	0 (0.0)	0 (0.0)	0 (0.0)
	Yes, live and work with	15 (36.6)	10 (38.5)	5 (33.3)	3 (25.0)	2 (66.7)
	Yes, do not live and work with	0 (0.0)	0 (0.0)	0 (0.0)	0 (0.0)	0 (0.0)
	Not sure	0 (0.0)	0 (0.0)	0 (0.0)	0 (0.0)	0 (0.0)
	Missing	4	3	1	1	0
Close contact with positive test	No	22 (52.4)	10 (38.5)	12 (75.0)	11 (84.6)	1 (33.3)
	Yes, live with	1 (2.4)	1 (3.9)	0 (0.0)	0 (0.0)	0 (0.0)
	Yes, work with	8 (19.0)	7 (26.9)	1 (6.3)	0 (0.0)	1 (33.3)
	Yes, live and work with	0 (0.0)	0 (0.0)	0 (0.0)	0 (0.0)	0 (0.0)
	Yes, do not live and work with	0 (0.0)	0 (0.0)	0 (0.0)	0 (0.0)	0 (0.0)
	Not sure	11 (26.2)	8 (30.8)	3 (18.8)	2 (15.4)	1 (33.3)
	Missing	3	3	0	0	0

Fifty-percent (8/16) of all cases reported presence of symptoms compatible with COVID-19, and three reported three or more symptoms. Symptoms included loss of taste (7/8; 87.5%), dry or productive cough (3/8; 37.5%), fever (3/8; 37.5%) and shortness of breath (5/8; 62.5%). No cases and 11.5% (3/26) of non-cases reported working with a symptomatic contact, and 6.3% (1/16) of cases and 26.9% (7/29) of non-cases reported working with a positive contact; however, all cases in the COVID-OUT study who reported having symptoms indicated that they stopped working when they were symptomatic. One (3.6%; 1/28) non-case and none of the cases had received a COVID-19 vaccine prior to the outbreak. After the start of the outbreak, 12 (44.4%; 12/27) non-cases and 5 (31.3%) cases had received a COVID-19 vaccine dose (vaccine type not reported) (Table 4).

## Discussion

The warehousing sector, including storage and distribution centres, has been significantly impacted by workplace outbreaks of COVID-19^2^. Here, we investigated an outbreak of SARS-CoV-2 infections at a storage and distribution centre in England, which occurred during the phased exit from the third national lockdown and during a period of decreasing infection rates in the local area. The attack rate (26.5%) for this outbreak suggests an increased risk of infection compared to the local community case rates, as has been observed for other workplace outbreaks^22,27^.

The company had implemented infection control measures before the outbreak, including signs and floor markings to promote 2m social distancing, enhanced cleaning measures (e.g., touch point cleaning of workspaces conducted every 3 h, mandatory use of face coverings whilst walking around the building, dividers installed at workstations, additional welfare facilities (e.g., canteens and toilets), and work-from-home accommodations for office workers. Following the outbreak, the company introduced further control measures, including additional cleaning and mandatory face covering use at all times. The overall adherence of workers to infection control measures at this site was observed to be high by occupational hygienist assessments as part of this study. However, assessments made by occupational hygienists during the site visits may not fully depict worker behaviours during the outbreak and may be subject to the Hawthorne effect [reviewed in^28^], with workers’ awareness of observation and social desirability to comply with infection control measures potentially overestimating adherence to control measures during these visits. The on-site assessments conducted following the outbreak also concluded that environmental factors, such as humidity, temperature and ventilation, were within normal parameters.

Although the company implemented enhanced cleaning measures prior to and during the outbreak, and their own investigations did not indicate any work area specific clustering of cases, our environmental surface sampling identified SARS-CoV-2 RNA on many surfaces and high-touch points across the site. While the levels of RNA detected were low, with no sample identified with a Ct value < 32.9, the high frequency of RNA detection at this site suggests multiple contamination events, which is in line with the high number of positive cases in the workforce identified by screening programmes. The seven samples with Ct values of ≤ 35.0 were subjected to WGS; however, no SARS-CoV-2 genome was identified within established quality control parameters, indicating significant degradation of the nucleic acid. Although this indicates that the material sampled was unlikely to present a fomite transmission risk at the time of sampling, it is not possible to conclude whether these surfaces contained infectious material closer to the original contamination events. A report of surface sampling results was provided to the site within one week of sampling being conducted with a recommendation to review and strengthen cleaning practices in both work and communal areas across the site.

The COVID-19 pandemic heightened financial concerns and saw an increase in adverse mental health implications^29^, with essential workers having increased risk of anxiety compared to their non-essential worker counterparts^30^. Of the approximate 1150 workers based at this outbreak investigation site, half were agency workers. The precarious contracts of such workers may further contribute to their financial uncertainties and anxiety. Furthermore, although most workers completing the COVID-OUT questionnaire were on permanent work contracts, this sample still noted concerns about potential detrimental financial and employment-related impacts of self-isolation. Strikingly, approximately one-quarter of respondents reported working with a positive or symptomatic contact, which may reflect a culture of presenteeism. To ease the concerns related to self-isolation, workplaces may consider encouraging clear communication about the support available to workers and the development of enhanced and equitable sick leave policies.

The questionnaire data provide evidence that workers had limited social activities and contacts outside of the workplace, in line with the recommendations of the phased exit from the government lockdown restrictions. Although the questionnaires were anonymised, the responses were self-reported and social desirability bias could have led to an overestimation of reported adherence to infection control measures both in the community and the workplace. While two previous studies investigating COVID-19-related intervention surveys found only a limited impact of social desirability bias on the estimates of compliant behaviours^31,32^, future observational studies could further mitigate the risk of social desirability bias by adopting “guilt-free strategies” for questionnaire design (e.g., including short preambles to mitigate potential concerns about reporting non-compliance)^33^. In addition, behavioural modifications may have also arisen in response to the outbreak itself.  Another limitation of our study was the very low worker participation rate, which limits the generalisability of the questionnaire findings within this workplace, as well as to other similar workplaces. Additionally, the small sample size precluded the precise estimation of relative risks at the individual level. Selection bias also remains a concern, as the study sample included an over-representation of workers on permanent contracts. Enhanced engagement with company supervisors on the production floor about the study might improve response rates and encourage worker participation for more detailed analysis to be conducted.

## Conclusion

This study shows that even with robust implementation of recommended public health measures, workplaces with large workforces, where workers have a high number of contacts, can be at risk of SARS-CoV-2 outbreaks. Workplace risk assessments remain an essential tool for assessing and controlling occupational COVID-19 risks. Importantly, risk assessments must be tailored to a given worksite and consider the workplace as a heterogenous collection of distinct work environments rather than a single entity. Enhanced infection control measures and tailored interventions should be detailed for each distinct work area. Such assessments must also be kept under review and amended with changes in local transmission rates. In addition to targeted workplace infection control measures, enhanced sick leave policies should be considered to support workers’ compliance to self-isolation requirements. Given the potential to perpetuate transmission into the local community, the prevention of outbreaks in work sectors with large workforces, especially those that remain open during lockdowns, such as storage and distribution centres, remains of high public health importance. While it is not possible to completely prevent cases of disease within a workforce during a national pandemic, it is imperative for workplaces to implement cost-effective transmission control measures based on risk assessments and to effectively minimise the on-site attendance of potential infectious workers through enhanced and equitable sick pay policies, where possible. Further research will be needed to better understand how working conditions and socioeconomic factors, such as crowded workspaces, insecure employment and income inequality, contribute to the increased infection risk among workers from essential work sectors. The improved evidence base in these areas can protect vulnerable populations, ensure the continued operations of essential work sectors during pandemics, and inform preparedness for future public health emergencies.

### Supplementary Information

Supplementary Information.

## Data Availability

The data that support the findings of this study are available on reasonable request from the corresponding author. The data are not publicly available due to privacy or ethical restrictions.
