# Supplementary material for: A SARS-CoV-2 outbreak investigation at a storage and distribution centre in England: an assessment of worker- and workplace-related risk factors
Source: Sci Rep. 2024 Apr 26;14:9561. doi: 10.1038/s41598-024-60194-4 (PMC11053053; doi:10.1038/s41598-024-60194-4)
Supplement: Supplementary file 1 — Supplementary Information. [file 41598_2024_60194_MOESM1_ESM.pdf]

Supplementary information

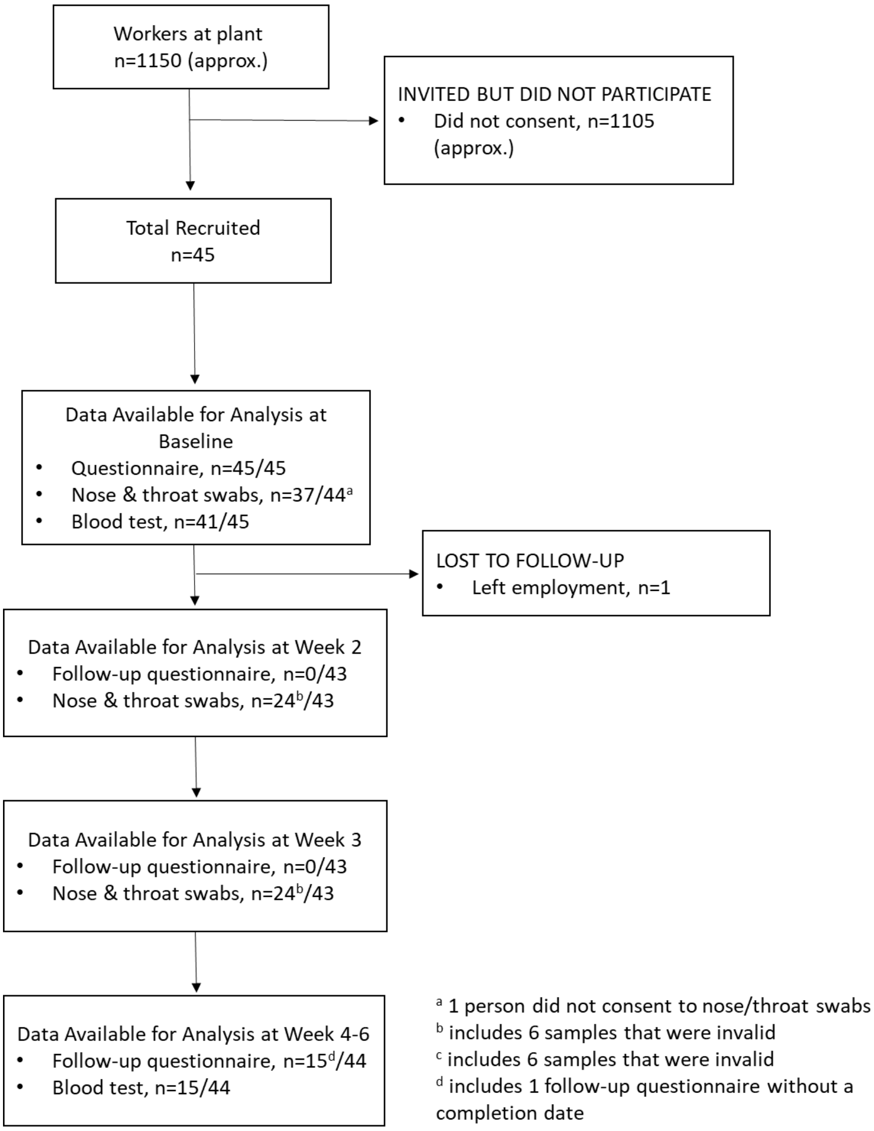

Figure S1. Flow diagram of workers from a storage and distribution site participating in COVID-OUT.

Table S1. Details of positive and suspected positive SARS-CoV-2 RNA surface samples taken from various locations following an outbreak at a storage and distribution centre – England, UK.

Site area	Location in area	Mean Ct value[a]	Estimated copies per cm[2b]
Building 1 Main canteen	Table + chair	33.2	1158
Building 1 Main canteen	Table + chair	37.2	34
Building 1 Main canteen	2x Vending machines	35.6	180
Building 1 Main canteen	Fridge + hot water lever	36.9[c]	47
Building 1 Break-out area	Table + chair	37.4	41
Building 1 Break-out area	Front of lockers L1, L2, L3, L4	37.1	96
Building 1 Break-out area	Top of L1 locker	34.7	806
Building 1 Break-out area	Front of lockers L5, L6, L7, L8	35.2	507
Building 1 Turnstile	Turnstile	35.1	574
Building 1 3rd Floor	Packing bench B1 + mouse	34.6	885
Building 1 3rd Floor	Packing bench B2 + mouse	32.9	3628
Building 1 3rd Floor	Supervisor Desk D1	34.9	687
Building 1 2nd Floor	Packing bench B3 + mouse + label print + scanner handle	36.1	232
Building 1 2nd Floor	Unlabelled packing bench B4 + scanner + mouse	37.9[c]	50
Building 1 2nd Floor	Trolley T1	36.1	235
Building 1 2nd Floor	Trolley T2	36.6	152
Building 1 1st Floor	Station 2 + mouse + scanner + stool	35.7	333
Building 1 1st Floor	Packing bench B5 + mouse	37.7[c]	55
Building 1 Ground Floor	Office desk + chair handles	36.8	182
Building 1 Ground Floor	Office desk + chair handles	35.3	970
Building 1 Ground Floor	Desk D2 + mouse + scanner	37.6[c]	81
Building 1 Ground Floor	Desk D3 + mouse + scanner	37.8	127

Building 1 Ground Floor	Desk D4 + scanner + brush + roller	38.0[c]	110
Building 1 Ground Floor	Bench B6 + scanner + brush + roller	37.6[c]	145
Building 1 Ground Floor	Bench B7 + scanner + brush + roller	37.7[c]	55
Building 1 Ground Floor	Bench B8 + scanner + brush + roller	36.1[c]	235
Building 1 Ground Floor	Bench B9 + scanner + brush + roller	37.4	182
Building 1 Ground Floor	Bench B10 + scanner + brush + roller + mouse	36.6	351
Building 1 Ground Floor	Bench B11 + scanner + brush + roller + mouse	37.2[c]	85
Building 1 Ground Floor	Screen S3	36.0	643
Building 1 Ground Floor Male Toilets	Bench B12 in toilets	37.9[c]	25
Building 1 Ground Floor Male Toilets	Cupboard + tool handles	38.0[c]	58
Building 1 Ground Floor Male Toilets	Cubicle C1 flush button + inner lock	35.0	884
Building 2 Ground Floor	Bench B13 + mouse + scanner	36.7	145
Building 2 Ground Floor	Bench B14 + mouse + scanner	37.4[c]	144
Building 2 1st Floor	Cabinet	34.7	792
Building 2 1st Floor	Screen behind S1	38.0[c]	111
Building 2 1st Floor	Screen behind S2	37.8[c]	123
Building 2 1st Floor	Bench B15 + mouse + scanner	35.5	503

[a]Mean Ct value for the N gene. [b]Extrapolation from copies per reaction to copies per sample collected based on the dilution factor, then divided by recorded sampling area. [c]Sample identified as suspected positive, defined as a sample with a single replicate testing positive for at least one target. Abbreviations: Severe acute respiratory syndrome coronavirus 2 (SARS-CoV-2), Ribonucleic acid (RNA), Real-time polymerase chain reaction (RT-PCR), Crossing threshold (Ct), Nucleocapsid (N).
